# Interfacial Redox-Driven Crystallization on MXene Enables Ultrasensitive Hg^2+^ Detection

**DOI:** 10.1021/acsami.6c05126

**Published:** 2026-06-10

**Authors:** Jiaxing Sun, Hanlin Jiang, Kartikey J. Chavan, Juwon S. Afolayan, Carole C. Perry, Xianfeng Chen

**Affiliations:** a Department of Physics, School of Science and Technology, 6122 Nottingham Trent University, Nottingham NG11 8NS,United Kingdom; b Department of Chemistry, School of Science and Technology, 6122 Nottingham Trent University, Nottingham NG11 8NS,United Kingdom

**Keywords:** MXene, redox-driven crystallization, Fabry−Pérot interferometer, nanophotonic sensor, heavy metal detection, trace-level sensing

## Abstract

Mercury contamination in water poses a serious threat to public health and ecosystems, demanding rapid and ultrasensitive detection at trace levels. We present, for the first time, an MXene-integrated fiber-optic Fabry–Pérot interferometer (MXene-FFPI) that demonstrates a mechanism-guided sensing platform for ultrasensitive Hg^2+^ detection at trace concentrations. High-quality mono- and bilayer Ti_3_C_2_T_*x*_ MXene nanosheets were synthesized via a minimally intensive layer delamination method, serving as efficient optical transducers and signal amplifiers. The sensing principle is based on interfacial redox-driven crystallization, in which Hg^2+^ ions are captured by MXene and converted into crystalline Hg_2_Cl_2_ nanoclusters, translating chemical interactions into amplified optical signals through modulation of the refractive index of the intracavity medium. Owing to the strong adsorption affinity and catalytic activity of MXene, chemical events are directly transduced into measurable interferometric responses. The MXene-FFPI exhibits an ultrahigh sensitivity of 5 pm/nM and a trace-level limit of detection (LOD) of 0.2 nM (0.04 ppb), more than 2 orders of magnitude lower than the WHO guideline limit for mercury in drinking water. This work establishes an MXene-interferometer nanophotonic architecture that converts interfacial reactions into amplified signals, providing a promising platform for ultrasensitive environmental monitoring and biomedical sensing.

## Graphical abstract

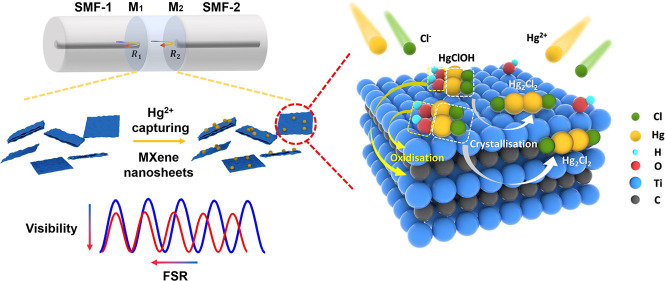

## 1 Introduction

The rapid expansion of human activities, coupled with industrialization and population growth, has led to increased heavy metal pollution in aquatic ecosystems. Mercury (Hg) is particularly hazardous and is listed by the World Health Organization (WHO) as one of the top ten chemicals of greatest public health concern.^1,2^ Due to its bioaccumulation and biomagnification in the human body, prolonged exposure to mercury can cause severe health effects, including central nervous system damage, birth defects, and chromosomal alterations.^3,4^ In aqueous environments, mercury primarily exists as Hg^2+^, with a WHO guideline value of 6 ppb in drinking water.^5^ These risks highlight the urgent need for sensitive, rapid, and portable Hg^2+^ detection methods. Conventional analytical techniques such as atomic fluorescence spectrometry,^6^ atomic absorption spectrometry,^7^ and inductively coupled plasma mass spectrometry^8^ provide high accuracy but rely on bulky, expensive instruments and involve complex, time-consuming sample preparation, limiting their practical applicability. Such limitations underscore the need for rapid, portable, and ultrasensitive sensing strategies.

Nanomaterial-based sensing platforms have emerged as promising alternatives due to their high surface-to-volume ratios and tunable surface chemistry.^9,10^ Mechanisms such as target-induced aggregation, antiaggregation, and surface functionalization allow nanomaterials to transduce chemical and biological interactions into measurable optical signals with high specificity and selectivity.^11,12^ MXenes,^13,14^ a rapidly expanding family of two-dimensional (2D) transition metal carbides, nitrides, and carbonitrides, have attracted considerable attention for applications in energy storage,^15^ optoelectronics,^16^ gas sensing,^17^ and biomedical detection.^18^ Since the first report in 2011, MXene has been synthesized via selective etching of the A-layer from MAX phase precursor (M_n+1_AX_n_), producing loosely stacked 2D layers with the general formula M_n+1_X_n_T_*x*_ (n = 1–4), where M is a transition metal, A is an element from group IIIA or IVA, X is carbon and/or nitrogen, and T_*x*_ represents surface termination groups (−O, −OH, −F, etc.).^19,20^ Removal of A-layer atoms weakens interlayer interactions, enabling exfoliation into mono- or bilayer nanosheets.^21^ The abundant surface terminations introduced during etching and delamination (−OH, −F, −Cl) provide chemically active sites for adsorption and redox interactions with heavy metal ions.^22^ This unique surface chemistry, combined with high electrical conductivity and strong optical responsiveness, makes MXene particularly attractive for Hg^2+^ sensing applications.^23,24^

Optical fiber technologies provide compact and highly sensitive platforms for transducing interfacial chemical interactions into measurable optical signals.^25,26^ Fiber-optic sensors are widely valued for their small footprint, immunity to electromagnetic interference, cost-effectiveness, rapid response, and high sensitivity.^27,28^ Various fiber-optic architectures have been developed for chemical and biological sensing, including fiber-optic Fabry–Pérot interferometers (FFPIs),^29,30^ tapered fibers,^31^ and fiber gratings.^32–34^ Among these, FFPIs are particularly attractive owing to their simple fabrication, structural versatility, and high measurement precision across diverse sensing modalities. Recent studies have explored MXene-integrated fiber-optic platforms for applications such as ultrafast nonlinear optical switching and refractive index (RI) sensing. Although these approaches demonstrate the potential of MXene to enhance optical performance, they are predominantly limited to passive RI modulation.^35–37^ More importantly, the rich surface chemistry, adsorption affinity, and interfacial reactivity of MXene remain largely underexploited in fiber-optic sensing. Meanwhile, achieving high sensitivity at ultralow analyte concentrations continues to be a major challenge for conventional fiber-optic sensors, motivating the integration of advanced functional nanomaterials with interferometric platforms to directly transduce interfacial chemical processes into amplified optical responses.

In this work, we report the first MXene-functionalized FFPI (MXene-FFPI) for ultrasensitive Hg^2+^ detection. Ti_3_C_2_T_*x*_ MXene nanosheets were synthesized via a minimally intensive layer delamination (MILD) method, producing mono- and bilayer structures with abundant surface terminations that provide chemically active sites for heavy metal adsorption and redox reactions. Integrated within the Fabry–Pérot cavity, the MXene nanosheets function as both optical transducers and signal amplifiers, directly coupling nanoscale interfacial reactions to measurable optical responses. Specifically, interfacial redox-driven crystallization captures Hg^2+^ ions on the MXene nanosheets and converts them into crystalline Hg_2_Cl_2_ nanoclusters, modulating the local RI within the cavity and amplifying optical interference signals at subnanomolar concentrations. By combining the unique interfacial chemistry of MXene with the intrinsic sensitivity of the FFPI, the MXene-FFPI achieves ultrasensitive detection, establishes a new nanomaterials-interferometer architecture, and provides a promising route toward portable, high-performance sensing platforms for environmental and biomedical applications.

## 2 Experimental Section

### 2.1 Materials and Instrumentation

Ti_3_AlC_2_ MAX phase powder (325 mesh) was obtained from Carbon-Ukraine Ltd. (Ukraine). Lithium fluoride (LiF) powder (300 mesh), mercury standard solution for ICP analysis, and hydrochloric acid (HCl) were purchased from Sigma-Aldrich (UK). Deionized (DI) water produced using a pure water system (DUO, Avidity Science Ltd., UK) was used for the preparation of all aqueous solutions. Commercially available bottled natural mineral water (Volvic, France) was used as a representative drinking water sample for recovery experiments. UV-curable optical adhesive (NOA61 Thorlabs Inc., UK) was used to fabricate the FFPI device.

The morphology and structure of the MXene nanosheets were characterized by scanning electron microscopy (SEM) and energy dispersive spectroscopy (EDS) (JSM-7100F LV, JEOL Ltd., Japan), atomic force microscopy (AFM, Dimension Icon, Bruker Ltd., USA), and transmission electron microscopy (TEM; JEM-2100Plus, JEOL Ltd., Japan). X-ray diffraction (XRD) patterns were recorded using a powder diffractometer (Rigaku Ultima IV, Rigaku Corporation, Japan) with Cu Kα radiation operated at 40 kV and 50 mA over a 2θ range of 5°–80°. Raman spectra were acquired using a DXR Raman spectrometer (Thermo Fisher Scientific Inc., UK), while UV–vis absorption spectra were measured using a Cary 60 UV–vis spectrophotometer (Agilent Technologies Inc., UK). Optical characterization of the FFPI was performed using a super luminescent diode (SLD; S5FC1550S-A2, Thorlabs Ltd., UK) and an optical spectrum analyzer (OSA; MS9740B, Anritsu Ltd., Japan), with system alignment achieved using 3-axis flexure stages (NanoMax 300, Thorlabs Inc., UK).

### 2.2 Synthesis of Ti_3_C_2_T_*x*_ MXene Nanosheets

Ti_3_C_2_T_*x*_ MXene nanosheets are synthesized using a MILD method, as schematically illustrated in Figure 1. In this approach, a LiF/HCl mixture generates hydrofluoric acid (HF) in situ, enabling selective etching of aluminum (Al) layers from the Ti_3_AlC_2_ MAX phase (Figure 1a,d). Compared with direct use of concentrated HF, this method provides a milder and safer etching environment.^38,39^

**Figure 1:**
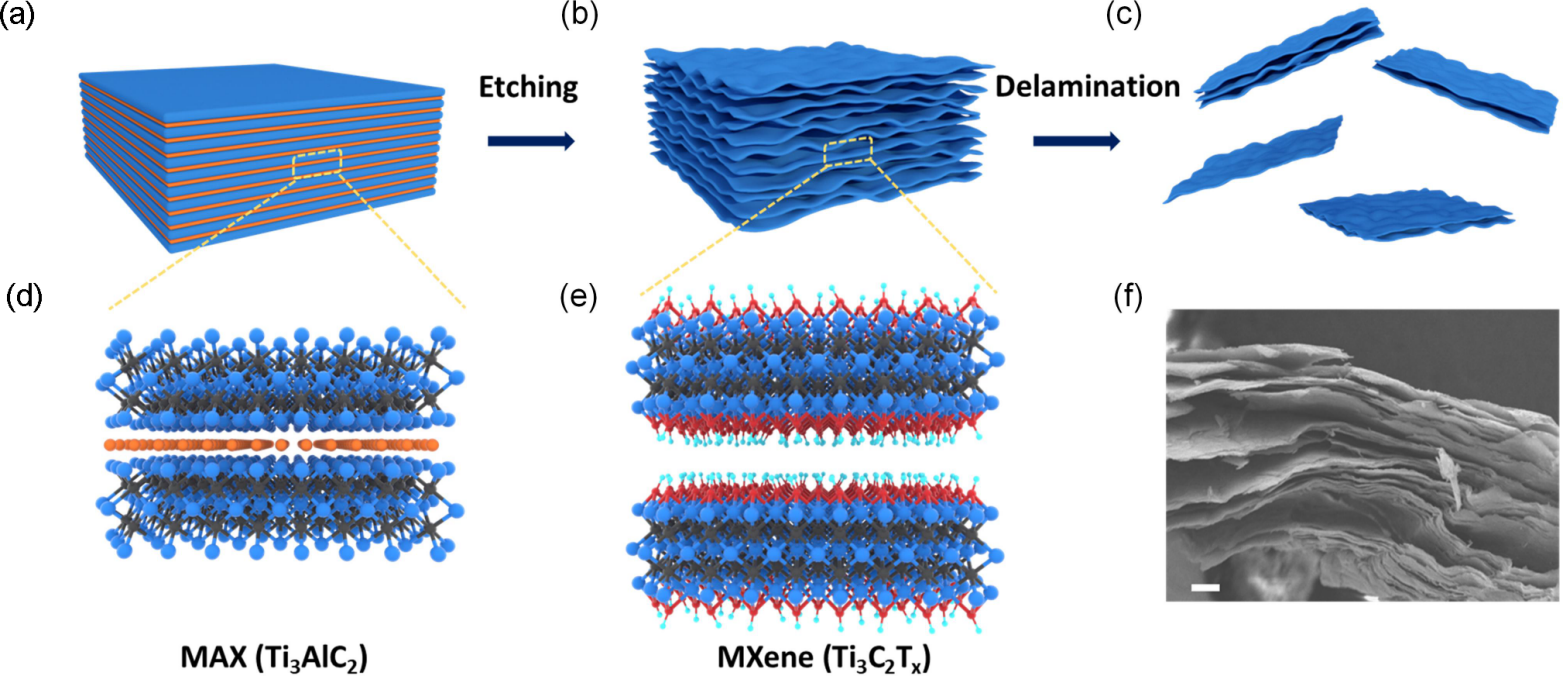
Schematical illustration of the synthesis and structural transformation of MXene (Ti_3_C_2_T_*x*_) from the MAX phase (Ti_3_AlC_2_). (a) Layered structure of the MAX phase. (b) Selective etching of the Al atomic layers to produce multilayer MXene, which is subsequently delaminated into mono- and bilayer MXene nanosheets (c). Corresponding atomic structures of Ti_3_AlC_2_ (d) and Ti_3_C_2_T_*x*_ (e), respectively. (f) SEM image of loosely stacked MXene nanosheets with an accordion-like morphology (scale bar: 10 μm).

Briefly, 2 g of LiF powder is dissolved in 35 mL of 10 M HCl to prepare the etching solution. Separately, 2 g of Ti_3_AlC_2_ MAX precursor is dispersed in 5 mL of DI water to form a homogeneous suspension, which is slowly added to the etching solution under gentle stirring. The mixture is maintained at 35 °C for 24 h to ensure selective Al removal (Figure 1b). After etching, the mixture is centrifuged at 3500 rpm for 5 min and washed repeatedly with DI water until the supernatant reaches pH ≈ 5.5, removing residual acids, suppressing overoxidation of MXene, and facilitating exfoliation. The resulting sediment, consisting predominantly of multilayer Ti_3_C_2_T_*x*_ (Figure 1e), is redispersed in 40 mL of DI water and exfoliated by vortex mixing for 1 h. Mono- and bilayer Ti_3_C_2_T_*x*_ nanosheets are obtained by collecting the supernatant after a second centrifugation at 3500 rpm for 1 h (Figure 1c). SEM imaging confirms the characteristic accordion-like morphology of the MXene (Figure 1f), indicating successful Al removal. This approach enables the preparation of high-quality Ti_3_C_2_T_*x*_ nanosheets under mild and controlled conditions.

The concentration of the Ti_3_C_2_T_*x*_ dispersion is determined gravimetrically by vacuum filtration of a known volume, followed by drying and weighing of the resulting film. UV–vis–NIR spectroscopy (Figure S1) reveals a linear relationship between absorbance at 755 nm and MXene concentration (inset, Figure S1), confirming good dispersibility and colloidal stability in aqueous media.

### 2.3 Mechanism of Interfacial Redox-Driven Crystallization and MXene Oxidation

The adsorption and catalytic reduction of mercury ions (Hg^2+^) by Ti_3_C_2_T_*x*_ MXene provide an effective pathway for Hg^2+^ capture and transformation.^24^ Figure 2 schematically illustrates the proposed interfacial redox reactions and crystallization processes. Hg^2+^ ions are adsorbed on the Ti_3_C_2_T_*x*_ surface, forming Cl–Hg–OH intermediates in chloride-containing media derived from the MILD synthesis (Section 2.2). These intermediates undergo homolytic cleavage, generating reactive radicals such as •OH and •HgCl. The •HgCl radicals dimerize to form crystalline Hg_2_Cl_2_, accompanied by energy release. The resulting Hg_2_Cl_2_ crystallites accumulate on the surfaces and edges of the MXene nanosheets, while the generated •OH radicals and released energy induce partial oxidation of Ti_3_C_2_T_*x*_, yielding TiO_2_/C nanocomposites.^24^

**Figure 2:**
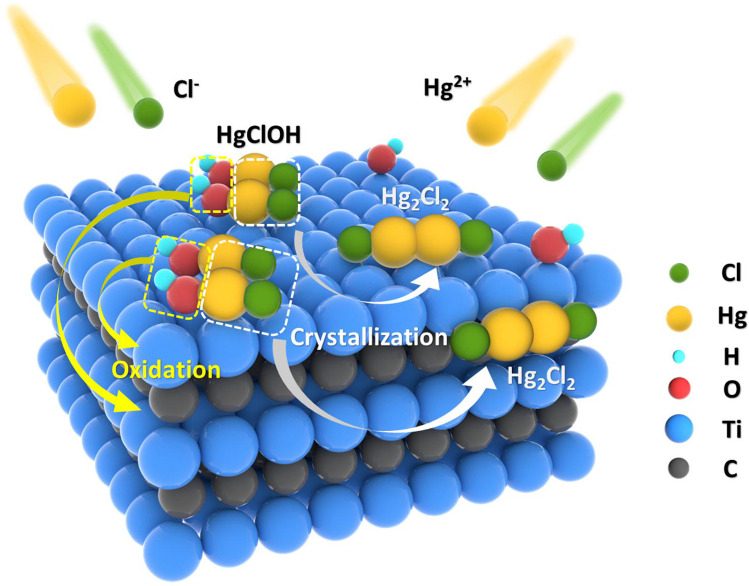
Schematic illustration of Hg^2+^ adsorption and interfacial redox-driven crystallization on Ti_3_C_2_T_*x*_ MXene, forming crystalline Hg_2_Cl_2_ nanoclusters, while the generated •OH radicals partially oxidize the MXene to TiO_2_/C nanocomposites.

This interfacial redox-driven process effectively translates subnanomolar variations in Hg^2+^ concentration into structural and chemical changes on MXene, forming the mechanistic basis for the amplified optical response observed in the MXene-FFPI. By coupling redox-induced crystallization with the intrinsic optical sensitivity of the FFPI, the platform achieves ultrasensitive, trace-level mercury detection.

### 2.4 FFPI-Based Sensing Principle

As illustrated in Figure 3, the FFPI is fabricated by inserting two single-mode fibers (SMFs) into a hollow capillary, where the finely cleaved fiber end facets act as two parallel mirrors (M1 and M2), thereby forming a F–P microcavity. The cavity length can be precisely adjusted using a pair of 3-axis stages. To enable direct interaction between the sensing medium and the analyte, a side-opening structure is introduced along the capillary, allowing the analyte solution to access the cavity region. After alignment, the fibers are fixed within the capillary using a UV-curable optical adhesive, producing a mechanically stable cavity with a fixed length.

**Figure 3:**
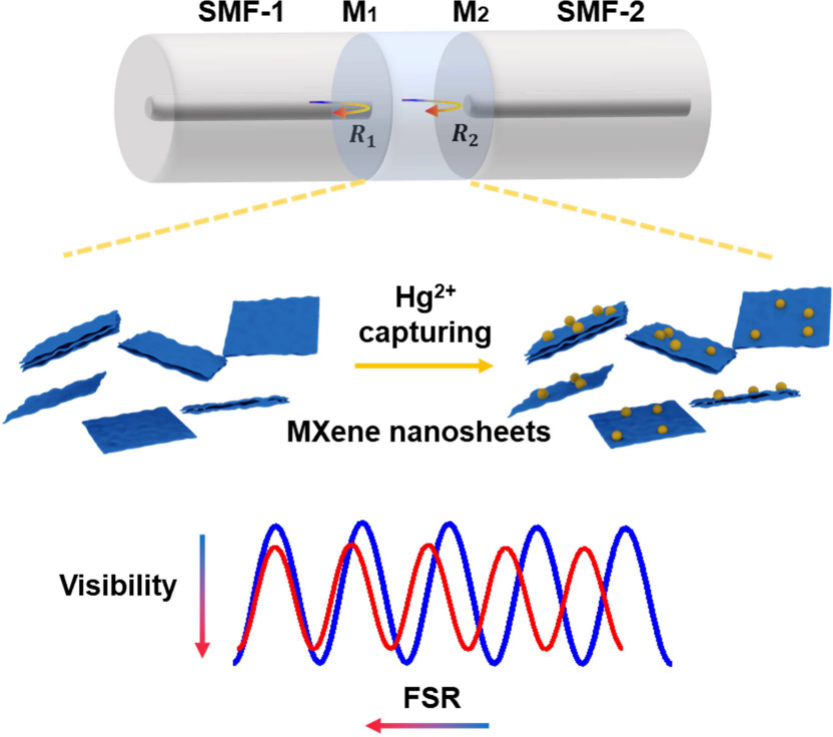
Schematic of the MXene-FFPI for Hg^2+^ detection.

Light launched from SMF-1 propagates into the microcavity and undergoes multiple reflections between the two parallel mirrors. The reflected light is subsequently collected by SMF-1 and recorded using an optical spectrum analyzer (OSA), producing a characteristic interference spectrum. For an FFPI, the total reflected irradiance *I*_*r*_ consists of contributions from all internal reflections within the cavity. Because the reflectivity of the fiber end facets is relatively low, high-order reflections contribute negligibly to the interference signal. Consequently, the reflected intensity can be approximated using a two-beam interference model:^40^

$$I_{r}=I_{1}+I_{2}+2\sqrt{I_{1}I_{2}}\cos\phi$$ 1

where *I*_1_ and *I*_2_ represent the reflected irradiance from M1 and M2 mirrors, respectively, and ϕ is the phase difference between the two interfering beams, given by

$$\phi =\frac{4\pi nL}{\lambda }$$ 2

here, *n* denotes the RI of the intracavity medium, *L* is the cavity length, and λ is the optical wavelength. Constructive interference occurs when the two reflected beams are in phase, whereas destructive interference arises when they are out of phase.

The wavelength spacing between adjacent interference fringes, defined as the free spectral range (FSR), can be expressed as

$$FSR=\frac{\lambda^{2}}{2nL}$$ 3

where λ is the central wavelength. For a given wavelength, the FSR depends on both the cavity length and the RI of the intracavity medium.

The strength of the interference pattern is characterized by the fringe visibility *V*, defined as

$$V=\frac{I_{\max}-I_{\min}}{I_{\max}+I_{\min}}$$ 4

where *I*_*max*_ and *I*_*min*_ represent the maximum and minimum intensities of the interference fringes, respectively. For an ideal symmetric cavity with mirror reflectivity *R*, the visibility can be approximated as

$$V=\frac{2\sqrt{R}}{1+R}$$ 5

Variations in the RI of the intracavity medium induce measurable changes in both the FSR and fringe visibility. As shown in Figure S2, both the FSR and visibility decrease when the intracavity medium is changed from air to water. When the cavity is filled with a Ti_3_C_2_T_*x*_ suspension containing Hg^2+^ ions, interfacial redox-driven crystallization occurs as Hg^2+^ ions are captured by MXene nanosheets and converted into Hg_2_Cl_2_ nanoclusters. This crystallization process increases the effective local RI of the suspension, thereby modifying the optical path length within the cavity.^41^ In addition, the formation of Hg_2_Cl_2_ nanoclusters and interfacial adsorption layers introduces additional optical scattering and absorption within the cavity, which reduces the effective reflectivity at the fiber-liquid interfaces. As the Hg^2+^ concentration increases, these effects collectively lead to a progressive narrowing of the FSR and a reduction in interference fringe visibility. These optical responses are both theoretically predicted and experimentally observed in the interference spectrum obtained in this work.

## 3 Results and Discussion

### 3.1 Characterization of MXene and Hg^2+^@MXene

The morphology and elemental composition of Ti_3_C_2_T_*x*_ MXene were characterized using SEM and EDS. As shown in Figure 4a, the SEM image reveals the typical loosely packed, accordion-like structure of Ti_3_C_2_T_*x*_ MXene, indicating successful etching of the Al layers from the precursor MAX phase. EDS elemental mapping further confirms the uniform distribution of Ti, C, O, Cl, and F across the MXene sheets. The presence of O, Cl, and F indicates the existence of surface termination groups on the MXene.

**Figure 4:**
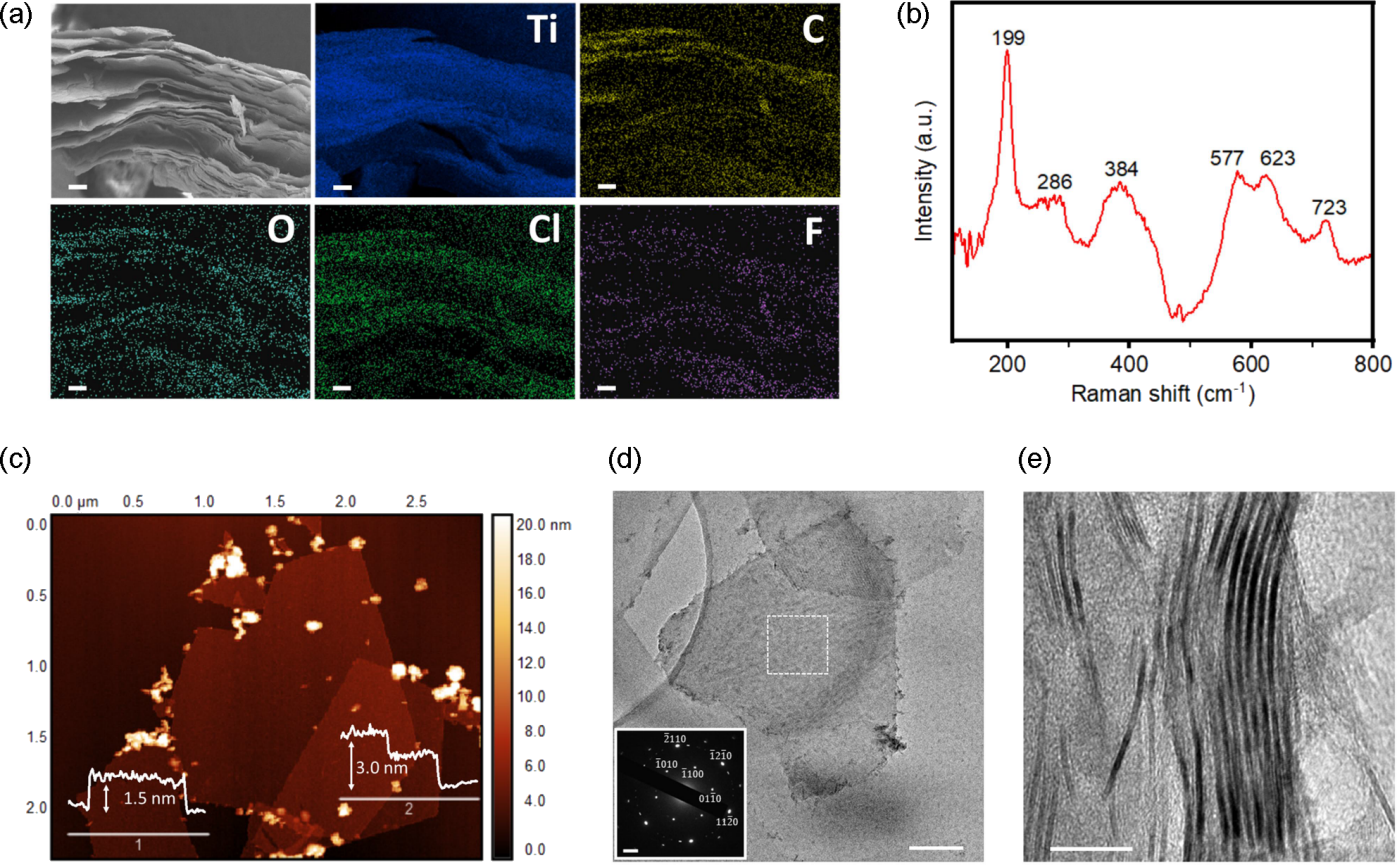
(a) SEM image of layered Ti_3_C_2_T_*x*_ MXene and corresponding EDS elemental maps of Ti, C, O, Cl, and F (scale bar: 10 μm). (b) Raman spectrum of Ti_3_C_2_T_*x*_ MXene. (c) AFM image of Ti_3_C_2_T_*x*_ nanosheets with height profiles extracted along the white lines (inset). (d) TEM image of a monolayer Ti_3_C_2_T_*x*_ nanosheet (scale bar: 200 nm) with the corresponding selected area electron diffraction pattern (inset, scale bar: 2 nm^–1^). (e) Cross-sectional TEM image showing mono- and bilayer Ti_3_C_2_T_*x*_ nanosheets (scale bar: 10 nm).

Figure 4b presents the Raman spectrum of Ti_3_C_2_T_*x*_ MXene, which exhibits characteristic vibrational modes. The prominent peak at 199 cm^–1^ is attributed to the out-of-plane A_1g_ mode, mainly associated with the vibrations of Ti, O, and C atoms. A broad peak centered at 723 cm^–1^ is also assigned to an A_1g_ mode, corresponding primarily to out-of-plane vibrations of carbon atoms. In addition, several in-plane vibrational modes (E_g_) are observed at 286, 384, 577, and 623 cm^–1^. The peaks at 286 cm^–1^ and 384 cm^–1^ are associated with in-plane E_g_ vibrations of surface functional groups bonded to titanium atoms, whereas the peaks at 577 cm^–1^ and 623 cm^–1^ mainly originate from in-plane carbon vibrations within the MXene lattice. These Raman features confirm the structural integrity and surface functionalization of the exfoliated Ti_3_C_2_T_*x*_ MXene.^42^

AFM was employed to determine the thickness of the exfoliated Ti_3_C_2_T_*x*_ nanosheets. As shown in Figure 4c, representative AFM height profiles of the exfoliated MXene nanosheets are presented. Profile 1 shows a height difference of ∼1.5 nm between the substrate and the nanosheet, which is consistent with the thickness of a typical monolayer Ti_3_C_2_T_*x*_ structure.^43^ In contrast, Profile 2 displays a step-like height increase to ∼3.0 nm, indicating the local vertical stacking of two exfoliated MXene monolayers. These results confirm the successful exfoliation of Ti_3_C_2_T_*x*_ into predominantly monolayer with lateral dimensions of approximately 1–2 μm.

TEM further corroborates these observations. Figure 4d shows a monolayer nanosheet with a lateral size of approximately 0.8–1.0 μm, consistent with the AFM measurements. The corresponding selected area electron diffraction (SAED) pattern (inset of Figure 4d) exhibits clear hexagonal symmetry of the lattice planes. The cross-sectional TEM image (Figure 4e) reveals both mono- and bilayer Ti_3_C_2_T_*x*_ nanosheets, in agreement with the AFM and TEM top-view observations.

To elucidate the crystallization of Hg^2+^ on MXene and its role in signal amplification, SEM, EDS, XRD, and Raman spectroscopy were employed to investigate the interfacial crystallization and oxidation processes. Figure 5a presents SEM images and corresponding EDS elemental maps of Ti_3_C_2_T_*x*_ following Hg^2+^ adsorption. Bright crystallites are observed on both the surfaces and edges of Ti_3_C_2_T_*x*_. EDS mapping further reveals the colocalization of Hg and Cl in these regions, consistent with the formation of Hg_2_Cl_2_ under chloride-containing conditions.^24^ These observations confirm the successful adsorption and interfacial crystallization of Hg^2+^ on the MXene. Similar phase-identification approaches have been employed to elucidate interfacial enhancement mechanisms in functional materials.^44^ Figure 5b shows the XRD patterns of pristine Ti_3_C_2_T_*x*_, and Ti_3_C_2_T_*x*_ after exposure to 500 and 1000 μM Hg^2+^ solutions. The characteristic diffraction peaks at 6.48° and 60.80° correspond to the (002) and (110) basal planes of Ti_3_C_2_T_*x*_, respectively. Following Hg^2+^ adsorption, additional diffraction peaks emerge at 2θ = 21.4° and 28.2°, which are assigned to Hg_2_Cl_2_, confirming the reduction and crystallization of Hg^2+^ on MXene. Furthermore, the intensities of these peaks increase with Hg^2+^ concentration, supporting the proposed crystallization-driven signal amplification mechanism. Raman spectra of Hg^2+^-adsorbed Ti_3_C_2_T_*x*_ (Figure 5c) reveal two additional bands at ∼155 and ∼503 cm^–1^, corresponding to anatase TiO_2_, together with the characteristic D and G bands of carbon at ∼1310 and ∼1580 cm^–1^, respectively. These features indicate partial oxidation of Ti_3_C_2_T_*x*_ into TiO_2_/C nanocomposites, likely mediated by reactive oxygen species generated during interfacial redox reactions. Similar combinations of complementary structural and spectroscopic characterization have been reported to verify chemically induced interfacial evolution and correlate it with mechanism-driven performance enhancement in functional materials.^45,46^

**Figure 5:**
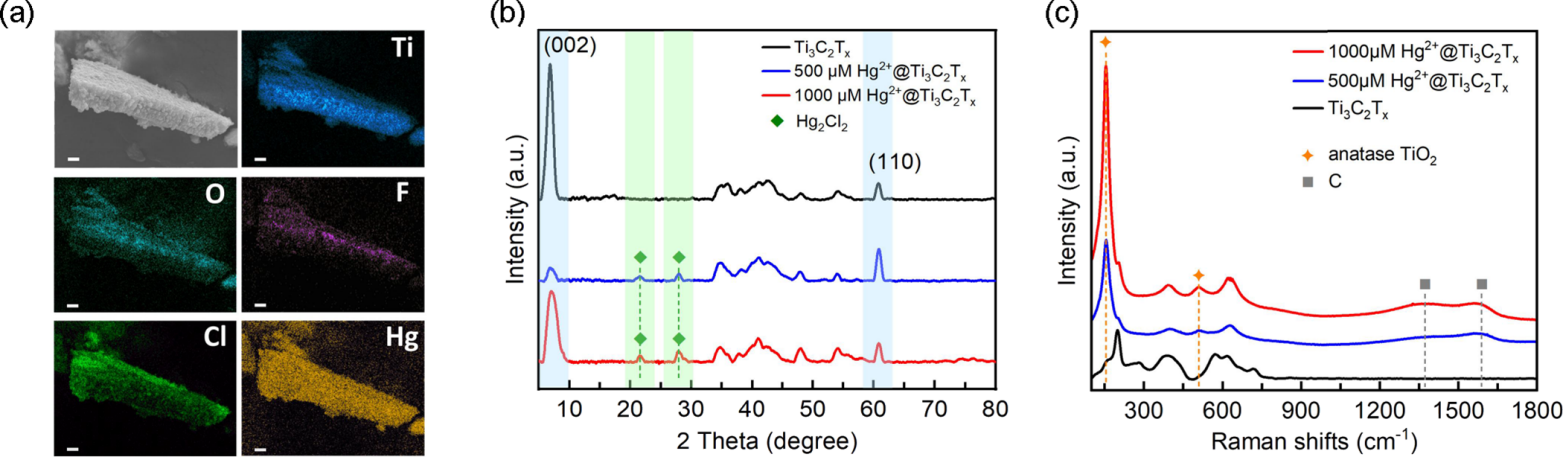
(a) SEM image of Ti_3_C_2_T_*x*_ MXene after Hg^2+^ adsorption, with corresponding EDS elemental maps of Ti, O, F, Cl, and Hg. The colocalization of Hg and Cl confirms successful Hg^2+^ uptake and interfacial crystallization on the MXene surface (scale bar: 2 μm). (b) Comparison of XRD patterns of pristine Ti_3_C_2_T_*x*_ and Ti_3_C_2_T_*x*_ after adsorption of 500 and 1000 μM Hg^2+^. (c) Raman spectra of pristine Ti_3_C_2_T_*x*_ and Ti_3_C_2_T_*x*_ after adsorption of 500 and 1000 μM Hg^2+^.

### 3.2 Hg^2+^ Detection Using a Conventional FFPI

The Hg^2+^ sensing performance of a conventional FFPI with a cavity length of 66 μm was first evaluated as a baseline for comparison. A 5000 μM Hg^2+^ stock solution was serially diluted to prepare test concentrations of 0, 0.001, 0.01, 0.1, 1, 10, 100, 500, 1000, 2500, and 5000 μM. Each solution was introduced into the FFPI cavity, which was thoroughly rinsed with DI water and dried between measurements to avoid cross-contamination. Prior to spectral acquisition, the solution was retained in the cavity for 1 min to ensure spectral stability.

The interference spectra recorded at different Hg^2+^ concentrations are shown in Figure 6a. As the Hg^2+^ concentration increases, the RI of the intracavity medium rises, resulting in a reduction of the FSR and observable shifts in the fringes. Figure 6b presents the FSR as a function of Hg^2+^ concentration, revealing two linear response regimes: 0–1.0 μM and 10–5000 μM, with the corresponding sensitivities of 28 pm/μM and 0.08 pm/μM, respectively. These results indicate that the conventional FFPI can detect Hg^2+^ over a broad concentration range, however its sensitivity at submicromolar levels remains limited, restricting its applicability for ultratrace detection.

**Figure 6:**
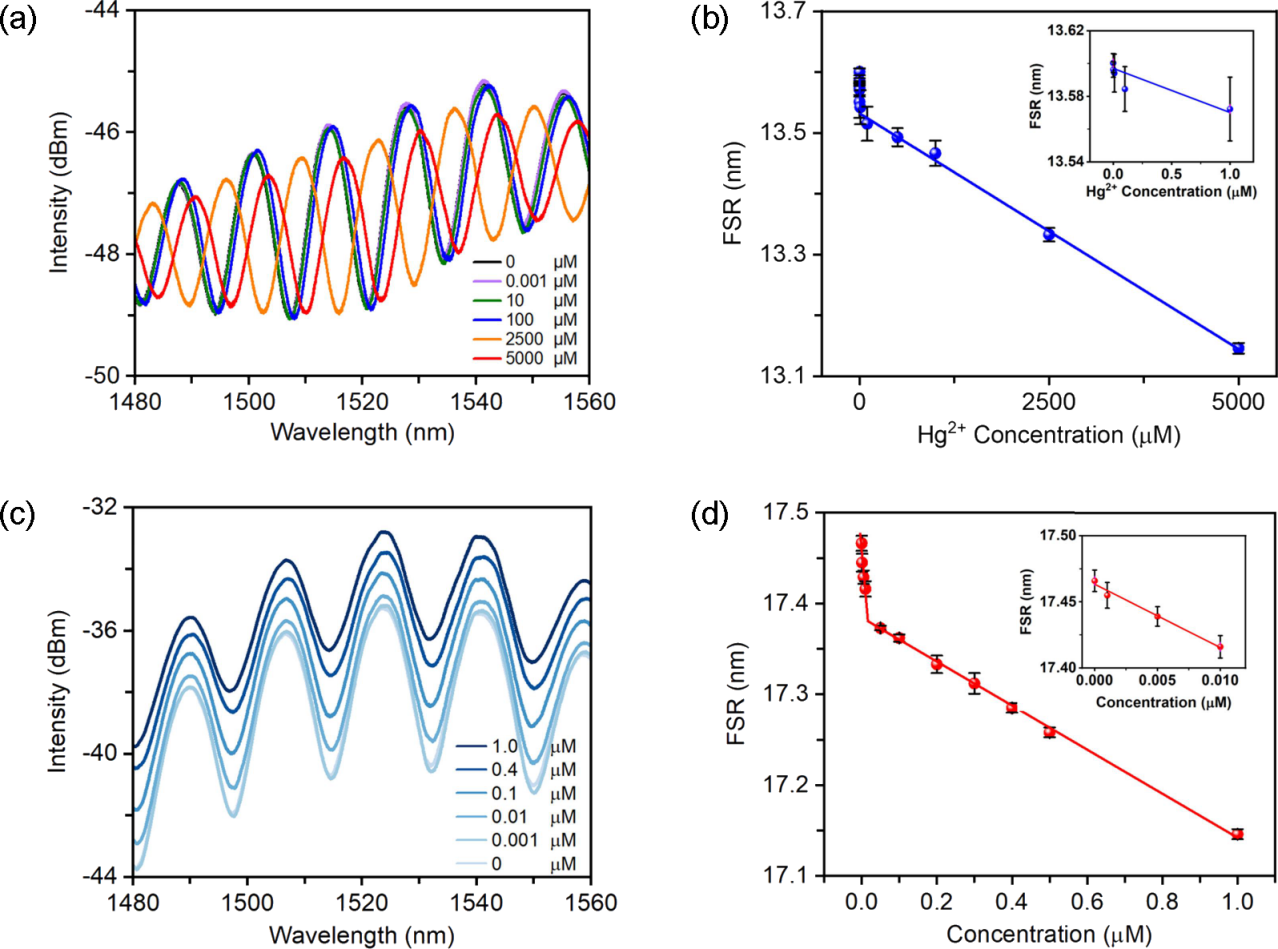
(a) Interference spectra of the conventional FFPI at different Hg^2+^ concentrations (selected spectra shown for clarity). (b) FSR as a function of Hg^2+^ concentration. Blue lines indicate linear calibration for the 0–1.0 μM and 10–5000 μM ranges (inset: low concentration range: 0–1.0 μM). (c) Interference spectra of the MXene-FFPI at different Hg^2+^ concentrations (selected spectra shown for clarity). (d) FSR versus Hg^2+^ concentration for the 0–0.01 μM and 0.05–1.0 μM regimes. Blue lines represent linear fits. (inset: low concentration range: 0–0.01 μM). All data in (b) and (d) are presented as mean ± s.d.

Fringe visibility was also analyzed. As the Hg^2+^ concentration increases, the visibility gradually decreases due to reduced Fresnel reflection at the fiber-liquid interfaces, which weakens multiple-beam interference and leads to less distinct fringes. As shown in Figure 7a, the conventional FFPI exhibits three linear visibility response regimes with sensitivities of 100%/mM (0–1.0 μM), 1%/mM (100–2500 μM), and 0.3%/mM (2500–5000 μM). Despite this response, the overall sensitivity remains insufficient for trace-level Hg^2+^ detection.

**Figure 7:**
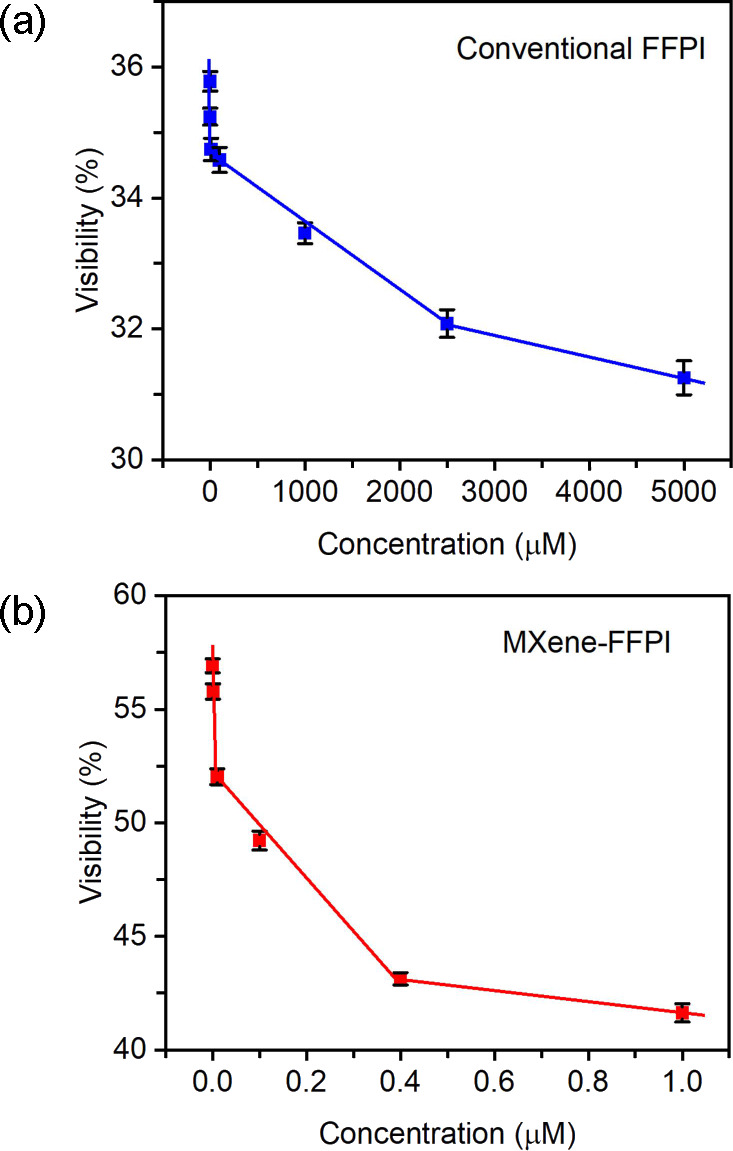
Interference fringe visibility versus Hg^2+^ concentration for (a) the conventional FFPI and (b) the MXene-FFPI. All data are presented as mean ± s.d.

### 3.3 Hg^2+^ Detection Using an MXene-FFPI

To overcome the above-mentioned limitations, MXene nanosheets were introduced into the FFPI cavity as optical transducers and signal-amplifying agents, enhancing the interferometric response of the sensor. A Ti_3_C_2_T_*x*_ suspension (0.5 mg/mL) was used as the functional buffer and mixed with Hg^2+^ solutions, taking advantage of the strong affinity of MXene toward Hg^2+^ ions. The suspension was divided into multiple aliquots and mixed with defined volumes of Hg^2+^ stock solution to prepare samples with final Hg^2+^ concentrations of 0, 0.001, 0.005, 0.01, 0.05, 0.1, 0.2, 0.3, 0.4, 0.5, and 1.0 μM. These mixtures were introduced into an FFPI cavity with a length of 50 μm. The cavity was rinsed with DI water and dried between measurements to avoid cross-contamination, and each sample was retained in the cavity for 1 min prior to spectral acquisition to allow sufficient Hg^2+^ adsorption and crystallization.

The interference spectra obtained from the MXene-FFPI are shown in Figure 6c. As the Hg^2+^ concentration increases, the RI of the intracavity medium rises, resulting in a progressive narrowing of the FSR and corresponding shifts in the interference pattern. The FSR exhibits two distinct linear response regimes with respect to Hg^2+^ concentration (Figure 6d). The sensitivity reaches 5000 pm/μM (i.e., 5 pm/nM, inset of Figure 6d) in the low-concentration regime (0–0.01 μM) and 240 pm/μM in the high-concentration regime (0.05–1.0 μM). Compared with the conventional FFPI, these values correspond to approximately 180-fold and 8.5-fold enhancements in sensitivity, respectively. Benefiting from the multifunctional role of MXene in promoting Hg^2+^ adsorption and interfacial redox-driven crystallization, the MXene-FFPI achieves a trace-level limit of detection (LOD) of 0.2 nM (0.04 ppb), assuming a high-precision optical interrogation system with a wavelength resolution of 1 pm.

The piecewise linear behavior in Figure 6b,d arises from the interplay between the intrinsic optical response of the FFPI interferometer and concentration-dependent interfacial evolution within the cavity. For MXene-FFPI, the low-concentration regime is governed by rapid Hg^2+^ adsorption and nucleation of Hg_2_Cl_2_ crystallites on Ti_3_C_2_T_*x*_, producing strong RI modulation, while the high-concentration regime reflects progressive crystallite growth and interfacial evolution supported by Figure 5, which reduces the incremental optical response per added analyte. The conventional FFPI shows a similar but weaker transition due to nonlinear RI variation of the intracavity medium.

Fringe visibility was also evaluated for the MXene-FFPI. As shown in Figure 7b, the visibility decreases with increasing Hg^2+^ concentration. This behavior arises from several factors. First, adsorption and interfacial crystallization of Hg^2+^ on Ti_3_C_2_T_*x*_ nanosheets increase the local RI, thereby reducing the effective Fresnel reflection at the fiber-liquid interfaces. Second, the formation of Hg_2_Cl_2_ nanoclusters and interfacial adsorption layers introduces additional optical scattering and weak absorption within the cavity. These effects reduce effective reflectivity, leading to a decrease in finesse and consequently less distinct interference fringes. The MXene-FFPI exhibits three linear visibility-based sensing regimes with significantly enhanced sensitivities of 488%/μM (0–0.01 μM), 22.8%/μM (0.01–0.4 μM), and 2.5%/μM (0.4–1.0 μM). Based on a power resolution of 0.01 dB, the corresponding LOD is estimated to be 0.47 nM (0.1 ppb).

Overall, incorporating Ti_3_C_2_T_*x*_ nanosheets significantly enhances the sensing performance of the MXene-FFPI. Strong interfacial affinity between MXene and Hg^2+^ facilitates adsorption and redox-driven crystallization, producing remarkable changes in the local RI and optical scattering. These effects amplify the interferometric responses, resulting in ultrahigh sensitivity and a trace-level LOD of 0.2 nM (0.04 ppb), over 2 orders of magnitude below the WHO guideline limit for mercury in drinking water. The simultaneous modulation of FSR and fringe visibility provides a dual-parameter sensing strategy, enhancing detection reliability and flexibility.

### 3.4 Repeatability, Selectivity, and Practical Applicability

The repeatability of the proposed sensing platforms was evaluated through five consecutive measurement cycles at the same Hg^2+^ concentration, with the FFPI cavity thoroughly rinsed with DI water between measurements. As shown in Figure S3, both the conventional FFPI and MXene-FFPI exhibited highly reproducible interference spectra and consistent FSR values over repeated cycles, confirming the excellent repeatability and operational stability of the sensing platforms.

To assess analytical selectivity, the MXene-FFPI (cavity length: 47 μm) was evaluated toward Hg^2+^ in the presence of representative metal ions, including Na^+^ and Mg^2+^. As shown in Figure 8a, Hg^2+^ produced a substantially greater FSR response than competing ions, whereas Na^+^ and Mg^2+^ induced only minor changes. As summarized in Table S1, the total FSR shift induced by Hg^2+^ over the concentration range of 0.1–1.0 μM reached 164 pm, which is approximately 4.6- and 3.6-fold greater than those induced by Mg^2+^ (36 pm) and Na^+^ (45 pm), respectively. These results demonstrate the strong selectivity toward Hg^2+^ detection, which is attributed to the preferential interfacial affinity and redox activity between Hg^2+^ ions and Ti_3_C_2_T_*x*_ nanosheets, promoting adsorption and crystallization-driven signal amplification.

**Figure 8:**
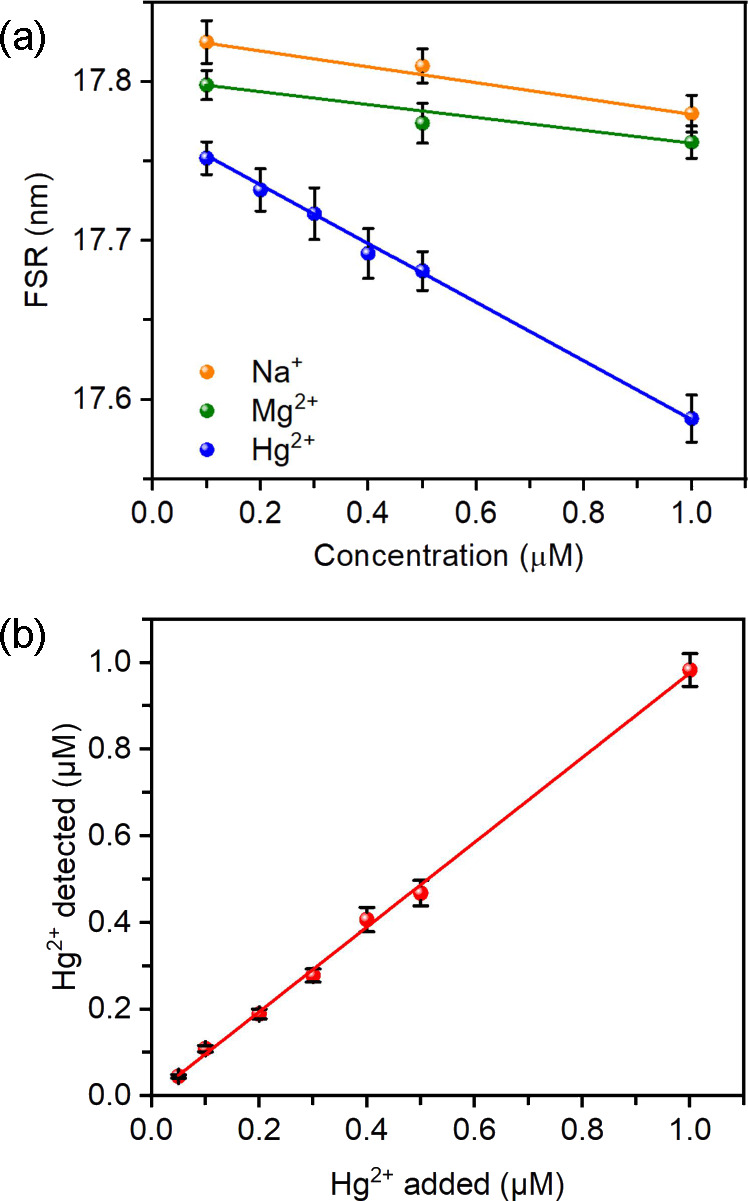
(a) Comparison of the FSR responses of the MXene-FFPI toward Hg^2+^, Mg^2+^, and Na^+^ over the concentration range of 0.1–1.0 μM. (b) Correlation analysis between the concentrations of added Hg^2+^ and detected Hg^2+^ in drinking water. Data are presented as mean ± s.d.

To validate practical applicability, commercially available bottled natural mineral water (Volvic, France) was employed as a representative drinking water matrix for Hg^2+^ recovery experiments. The interference spectra and corresponding FSR responses of the MXene-FFPI (cavity length: 47 μm) recorded in DI water and drinking water at different Hg^2+^ concentrations are presented in Figure S4a,b and Figure S4c,d, respectively. As shown in Figure 8b, the detected Hg^2+^ concentrations exhibit excellent agreement with the spiked concentrations in drinking water, demonstrating a strong positive correlation. As summarized in Table S2, recovery rates range from 90% to 109%, confirming the robustness, reliability, and quantitative accuracy of the proposed sensor in a realistic drinking water matrix. These results highlight the strong potential of the MXene-FFPI for practical environmental analysis, while future studies will further extend its application to more complex real water matrices, including tap water and river water.

## 4 Conclusions

In this work, we report the first MXene-FFPI as a high-performance platform for trace-level Hg^2+^ detection. By incorporating Ti_3_C_2_T_*x*_ nanosheets into the FFPI cavity, interfacial redox-driven crystallization of Hg^2+^ is effectively transduced into amplified optical signals, providing a robust mechanism for ultrasensitive detection. The high-quality mono- and bilayer Ti_3_C_2_T_*x*_ nanosheets serve as both efficient optical transducers and signal amplifiers, enabling pronounced modulation of intracavity RI and optical scattering. As a result, the MXene-FFPI achieves an ultrahigh sensitivity of 5 pm/nM and a trace-level LOD of 0.2 nM (0.04 ppb), more than 2 orders of magnitude lower than the WHO guideline limit for mercury in drinking water. In addition, simultaneous modulation of FSR and fringe visibility establishes a versatile dual-parameter sensing strategy, enhancing detection reliability while providing mechanistic insight and practical flexibility. Systematic evaluation of repeatability, selectivity, and practical applicability further confirms the robustness of the proposed MXene-FFPI for sensing applications. Overall, this work establishes a robust MXene-nanophotonic architecture that converts interfacial chemical reactions into amplified interferometric responses, highlighting its broad potential for ultrasensitive environmental monitoring and biomedical sensing.

## Supplementary Material



## Notes

UV–vis–NIR absorption spectra of Ti_3_C_2_T_*x*_ dispersion; reflection spectra of a cavity length-fixed FFPI with air and DI water as the intracavity media; repeatability of the conventional FFPI and MXene-FFPI evaluated by FSR; FSR responses of the MXene-FFPI to different analytes; MXene-FFPI for Hg^2+^ detection in DI water and bottled drinking water; recovery experiment data for the detection of Hg^2+^ in drinking water (PDF)

## References

[1] Bruno R., Mon M., Escamilla P., Ferrando-Soria J., Esposito E., Fuoco A., Monteleone M., Jansen J. C., Elliani R., Tagarelli A., Armentano D., Pardo E. (2021). Bioinspired metal-organic frameworks
in mixed matrix membranes for efficient static/dynamic removal of
mercury from water. Adv. Funct. Mater..

[2] WHO , 10 chemicals of public health concern; World Health Organization: Geneva, Switzerland. 2020.https://www.who.int/news-room/photo-story/detail/10-chemicals-of-public-health-concern (accessed 2026–03–05).

[3] McNutt M. (2013). Mercury and
Health. Science.

[4] Li B., Zhang Y., Ma D., Shi Z., Ma S. (2014). Mercury nano-trap
for effective and efficient removal of mercury (II) from aqueous solution. Nat. Commun..

[5] WHO , Guidelines for drinking-water quality: fourth edition incorporating the first and second addenda; World Health Organization: Geneva, Switzerland. 2022. https://www.who.int/publications/i/item/9789240045064 (accessed 2026–03–05). 35417116

[6] Campanella B., Onor M., Mascherpa M. C., D’Ulivo A., Ferrari C., Bramanti E. (2013). Determination of thiomersal by flow
injection coupled with microwave-assisted photochemical online oxidative
decomposition of organic mercury and cold vapor atomic fluorescence
spectroscopy. Anal. Chim. Acta.

[7] Shah A. Q., Kazi T. G., Baig J. A., Afridi H. I., Arain M. B. (2012). Simultaneously
determination of methyl and inorganic mercury in fish species by cold
vapour generation atomic absorption spectrometry. Food Chem..

[8] De
Souza S. S., Campiglia A. D., Barbosa F. (2013). A simple method for methylmercury, inorganic mercury and ethyl mercury
determination in plasma samples by high performance liquid chromatography–cold-vapor-inductively
coupled plasma mass spectrometry. Anal. Chim.
Acta.

[9] Oh S. H., Altug H., Jin X., Low T., Koester S. J., Ivanov A. P., Edel J. B., Avouris P., Strano M. S. (2021). Nanophotonic
biosensors harnessing van der Waals materials. Nat. Commun..

[10] Li G., Chen Y., Zhang X., Tang A., Yang H. (2025). Advances in
microfluidics-enabled dimensional design of micro-/nanomaterials for
biomedical applications: A Review. ACS Appl.
Mater. Interfaces.

[11] Afolayan J. S., Perry C. C. (2025). Functionalized AuNP-mycelial
composites as engineered
living materials for sustainable mercury remediation. RSC Sustain..

[12] Sun J., Zhou L., Li Z., He G., Mao H., Zhao J., Hunt J. A., Chen X. (2025). Perovskite-graphene
heterostructure biosensor integrated with biotunable nanoplasmonic
ternary logic gate for ultrasensitive cytokine detection. Adv. Sci..

[13] Naguib M., Kurtoglu M., Presser V., Lu J., Niu J., Heon M., Hultman L., Gogotsi Y., Barsoum M. W. (2011). Two-dimensional
nanocrystals produced by exfoliation of Ti_3_AlC_2_. Adv. Mater..

[14] Lim K. R. G., Shekhirev M., Wyatt B. C., Anasori B., Gogotsi Y., Seh Z. W. (2022). Fundamentals
of MXene synthesis. Nat. Synth..

[15] Lukatskaya M. R., Mashtalir O., Ren C. E., Dall’Agnese Y., Rozier P., Taberna P. L., Naguib M., Simon P., Barsoum M. W., Gogotsi Y. (2013). Cation intercalation
and high volumetric
capacitance of two-dimensional titanium carbide. Science.

[16] Han M., Maleski K., Shuck C. E., Yang Y., Glazar J. T., Foucher A. C., Hantanasirisakul K., Sarycheva A., Frey N. C., May S. J., Shenoy V. B., Stach E. A., Gogotsi Y. (2020). Tailoring electronic and optical properties of MXenes
through forming solid solutions. J. Am. Chem.
Soc..

[17] Arkoti N. K., Pal K. (2025). Ti_3_C_2_T_x_ MXene-derived metal–organic
frameworks for room temperature NO_2_ detection. ACS Appl. Mater. Interfaces.

[18] Jin P., Zheng W., Zhang Y. n., Zhou E., Li X., Zhao Y. (2025). Nb_2_C-MXene/UIO-66-COOH
hybrid-modified MZI sensor for
improved riboflavin detection. ACS Appl. Mater.
Interfaces.

[19] Naguib M., Mashtalir O., Carle J., Presser V., Lu J., Hultman L., Gogotsi Y., Barsoum M. W. (2012). Two-dimensional
transition metal carbides. ACS Nano.

[20] Hong X., Xu Z., Lv Z., Lin Z., Ahmadi M., Cui L., Liljeström V., Dudko V., Sheng J., Cui X., Tsapenko A. P., Breu J., Sun Z., Zhang Q., Kauppinen E., Peng B., Ikkala O. (2024). High-permittivity solvents
increase MXene stability and stacking order enabling ultraefficient
terahertz shielding. Adv. Sci..

[21] Naguib M., Mochalin V. N., Barsoum M. W., Gogotsi Y. (2014). 25th anniversary
article:
MXenes: a new family of two-dimensional materials. Adv. Mater..

[22] Martins T. S., Bott-Neto J. L., Oliveira O. N. (2024). Label- and redox
probe-free bioelectronic chip for monitoring vitamins C and the 25-hydroxyvitamin
D3 metabolite. ACS Appl. Nano Mater..

[23] Avinashi S. K., Mishra R. K., Singh R., Shweta, Rakhi, Fatima Z., Gautam C. R. (2024). Fabrication
methods, structural, surface morphology and biomedical applications
of MXene: a review. ACS Appl. Mater. Interfaces.

[24] Fu K., Liu X., Yu D., Luo J., Wang Z., Crittenden J. C. (2020). Highly
efficient and selective Hg­(II) removal from water using multilayered
Ti_3_C_2_O_x_ MXene via adsorption coupled
with catalytic reduction mechanism. Environ.
Sci. Technol..

[25] Yu H., Huang Z., Lamon S., Wang B., Ding H., Lin J., Wang Q., Luan H., Gu M., Zhang Q. (2025). All-optical
image transportation through a multimode fibre using a miniaturized
diffractive neural network on the distal facet. Nat. Photonics.

[26] Kohler L., Mader M., Kern C., Wegener M., Hunger D. (2021). Tracking Brownian
motion in three dimensions and characterization of individual nanoparticles
using a fiber-based high-finesse microcavity. Nat. Commun..

[27] Wu H., Chen P., Zhan X., Lin K., Hu T., Xiao A., Liang J., Huang Y., Huang Y., Guan B. (2024). Marriage of a dual-plasmonic interface
and optical microfiber for
NIR-II cancer theranostics. Adv. Mater..

[28] Tsogbayar D., Seo J., Hwang T., Kim Y., Park J., Oh S., Xu W., Lee H. S. (2025). Multimodal
double-helix fiber sensors for distinguishable
pressure and strain detection in wearable sensory applications. ACS Appl. Mater. Interfaces.

[29] Needham L. M., Saavedra C., Rasch J. K., Sole-Barber D., Schweitzer B. S., Fairhall A. J., Vollbrecht C. H., Wan S., Podorova Y., Bergsten A. J., Mehlenbacher B., Zhang Z., Tenbrake L., Saimi J., Kneely L. C., Kirkwood J. S., Pfeifer H., Chapman E. R., Goldsmith R. H. (2024). Label-free
detection and profiling of individual solution-phase molecules. Nature.

[30] Cheng H., Xiang C., Jin N., Kudelin I., Guo J., Heyrich M., Liu Y., Peters J., Ji Q.-X., Zhou Y., Vahala K. J., Quinlan F., Diddams S. A., Bowers J. E., Rakich P. T. (2025). Harnessing micro-Fabry–Pérot
reference cavities in photonic integrated circuits. Nat. Photonics.

[31] Mauranyapin N. P., Madsen L. S., Taylor M. A., Waleed M., Bowen W. P. (2017). Evanescent
single-molecule biosensing with quantum-limited precision. Nat. Photonics.

[32] Sun J., Jiang H., Chavan K. J., Coutts A. S., Chen X. (2025). Graphene oxide-functionalized
optical sensor for label-free detection of breast cancer cells. ACS Appl. Nano Mater..

[33] Liu D., Zhou L., Huang L., Zuo Z., Ho V., Jin L., Lu Y., Chen X., Zhao J., Qian D., Liu H., Mao H. (2021). Microfluidic
integrated capacitive biosensor for C-reactive
protein label-free and real-time detection. Anal..

[34] Juste-Dolz A., Delgado-Pinar M., Avella-Oliver M., Fernández E., Cruz J. L., Andrés M. V., Maquieira Á. (2022). Denaturing
for nanoarchitectonics: local and periodic UV-laser photodeactivation
of protein biolayers to create functional patterns for biosensing. ACS Appl. Mater. Interfaces.

[35] Uddin S., Debnath P. C., Kim H., Moon H., Koo C. M., Song Y.-W. (2024). Asymmetric laser
field interaction with MXene coated
on the side surface of optical fibers for ultrafast nonlinear switches. ACS Appl. Mater. Interfaces.

[36] Wang T., Zhu L., Kanda H. (2023). Ti_3_C_2_ MXene-TiO_2_ hybrid-modified
U-bend fiberoptic sensor for improved refractive index sensitivity
and ammonia detection. Sens. Actuators B: Chem..

[37] Chen Y., Ge Y., Huang W., Li Z., Wu L., Zhang H., Li X. (2020). Refractive index sensors
based on Ti_3_C_2_T_x_ MXene fibers. ACS Appl. Nano Mater..

[38] Ghidiu M., Lukatskaya M. R., Zhao M. Q., Gogotsi Y., Barsoum M. W. (2014). Conductive
two-dimensional titanium carbide ‘clay’ with high volumetric
capacitance. Nature.

[39] Downes M., Shuck C. E., McBride B., Busa J., Gogotsi Y. (2024). Comprehensive
synthesis of Ti_3_C_2_T_x_ from MAX phase
to MXene. Nat. Protoc..

[40] Wei T., Han Y., Li Y., Tsai H. L., Xiao H. (2008). Temperature-insensitive
miniaturized fiber inline Fabry-Perot interferometer for highly sensitive
refractive index measurement. Opt. Express.

[41] Krauz L., Páta P., Bednář J., Klíma M. (2021). Quasi-collinear
IR AOTF based on mercurous halide single crystals for spatio-spectral
hyperspectral imaging. Opt. Express.

[42] Sarycheva A., Gogotsi Y. (2020). Raman spectroscopy
analysis of the structure and surface
chemistry of Ti_3_C_2_T_x_ MXene. Chem. Mater..

[43] Guo T., Zhou D., Deng S., Jafarpour M., Avaro J., Neels A., Heier J., Zhang C. (2023). Rational design
of Ti_3_C_2_T_x_ MXene inks for conductive,
transparent films. ACS Nano.

[44] Zhang S., Gao Z., Zhang D., Lolupiman K., Limphirat W., Wu X., Qin J., Cao J. (2026). Hydrogen bond network induced interfacial
dipoles enhance built-in electric fields and ion transport in vanadium
oxide heterostructures. Energy Storage Mater..

[45] Zhang S., Lolupiman K., Zhang D., Gao Z., Chanajaree R., Zhang X., Cao J., Qin J. (2026). Dual-region synergistic
modulation and (101) facet engineering for highly reversible zinc
anodes. Adv. Powder Mater..

[46] Gao Z., Zhang S., Jin Y., Zhang D., Zhang X., Qin J., Cao J. (2026). Co-engineering
dual Helmholtz planes and (101) facet-selective
deposition for ultra-stable Zn metal anodes. J. Energy Chem..

