# Supplementary material for: Interfacial Redox-Driven Crystallization on MXene Enables Ultrasensitive Hg2+ Detection
Source: ACS Appl Mater Interfaces. 2026 Jun 10;18(24):34515–24. doi: 10.1021/acsami.6c05126 (PMC13307075; doi:10.1021/acsami.6c05126)
Supplement: Supplementary file 1 [file am6c05126_si_001.pdf]

Supporting Information

Interfacial Redox-Driven Crystallization on MXene Enables Ultrasensitive Hg^{2+} Detection

Jiaxing Sun[a, ‡], Hanlin Jiang[a, ‡], Kartikey J. Chavan[a], Juwon S. Afolayan[b], Carole C. Perry[b], Xianfeng Chen[a*]

[a] *Department of Physics, School of Science and Technology, Nottingham Trent University, Nottingham NG11 8NS, United Kingdom*
[b] *Department of Chemistry, School of Science and Technology, Nottingham Trent University, Nottingham NG11 8NS, United Kingdom*

As shown in **Figure S1**, the UV–Vis–NIR absorption spectrum of a $Ti_3C_2T_x$ nanosheet dispersion after sonication exhibits a characteristic shoulder at approximately 300 nm, which is commonly associated with freshly prepared $Ti_3C_2T_x$ dispersions. A broad absorption band centered at ~755 nm is also observed, which is attributed to the plasmonic response of $Ti_3C_2T_x$ nanosheets [R1, R2].

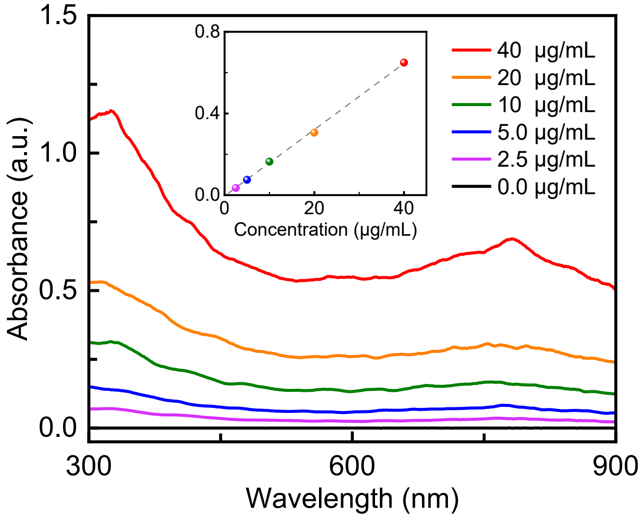

Figure S1. UV–Vis–NIR absorption spectra of freshly diluted $Ti_3C_2T_x$ MXene dispersions. Inset: Absorbance at 755 nm as a function of $Ti_3C_2T_x$ concentration (0, 2.5, 5.0, 10, 20, and 40 μg/mL), showing a linear relationship between absorbance and concentration.

* Corresponding author:
Email address: xianfeng.chen@ntu.ac.uk (X. Chen)
Tel: +44 115 8483118

Figure S2 presents the reflection spectra of a cavity length-fixed FFPI using air (red spectrum) and DI water (blue spectrum) as the intracavity medium. When the medium is changed from air (n = 1.0) to water (n = 1.333), two notable effects are observed. First, the FSR decreases, consistent with its inverse dependence on the RI of the cavity medium (Equation 3). Second, the fringe visibility decreases due to reduced Fresnel reflectivity at the fiber-medium interfaces associated with the higher RI of water, together with additional optical absorption loss in water.

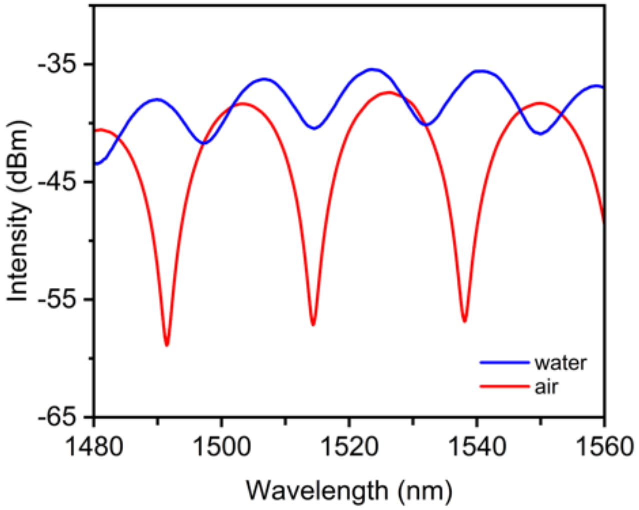

Figure S2. Reflection spectra of FFPI with air and water serving as the intracavity media.

Figure S3 presents the repeatability of both the conventional FFPI and MXene-FFPI over five consecutive measurement cycles, with the FFPI cavity thoroughly rinsed with DI water between measurements. For the conventional FFPI, a 100 μM Hg^{2+} solution was used as the analyte. As shown in Figure S3a,b, the interference spectra exhibit excellent overlap, while the extracted FSR values remain highly consistent across five cycles, with cycles 2–5 retaining 98–99% of the initial response. For the MXene-FFPI, a 0.2 μM Hg^{2+} solution was employed. Similarly, Figure S3c,d show highly reproducible interference spectra and consistent FSR values, with cycles 2–5 retaining 96–101% of the initial response. These results confirm the excellent repeatability and operational stability of the proposed sensing platforms.

After each measurement cycle, the cavity was thoroughly rinsed with DI water and dried prior to subsequent measurements. Measurements were resumed only after the interference spectrum returned to its original baseline position, indicating restoration of the cavity condition and suggesting that no measurable residual Hg/Cl-related deposits affecting the optical response remained within the cavity.

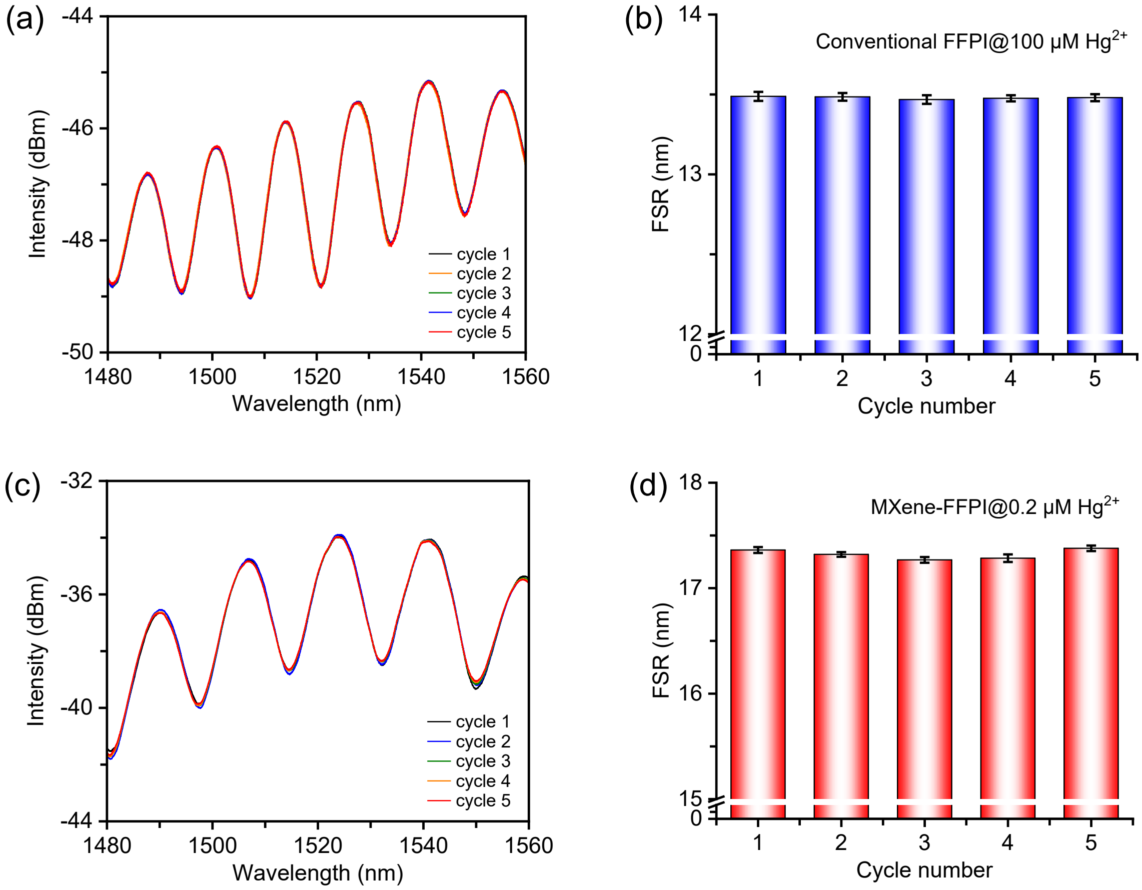

Figure S3. Repeatability of the conventional FFPI and MXene-FFPI evaluated by FSR. (a) Interference spectra of the conventional FFPI recorded over five repeated measurements at an Hg^{2+} concentration of 100 μM. (b) Corresponding FSR values over five cycles. (c) Interference spectra of the MXene-FFPI recorded over five repeated measurements at an Hg^{2+} concentration of 0.2 μM. (d) Corresponding FSR values over five cycles. Data in (b) and (d) are presented as mean ± s.d.

Table S1 compares the FSR responses and sensitivities of the MXene-FFPI (new cavity length: 47 μm) toward Hg^{2+}, Mg^{2+}, and Na^{+}. Over the concentration range of 0.1–1.0 μM, Hg^{2+} induces a total FSR shift of 164 pm, which is 4.6- and 3.6-fold greater than those induced by Mg^{2+} (36 pm) and Na^{+} (45 pm), respectively. The sensitivity toward Hg^{2+} reached 182 pm/μM, significantly exceeding those for Mg^{2+} (40 pm/μM) and for Na^{+} (50 pm/μM). These results demonstrate the strong selectivity of the MXene-FFPI toward Hg^{2+} detection, which is attributed to the preferential interfacial affinity and redox-driven crystallization of Hg^{2+} on MXene.

Table S1. FSR response and sensitivity of the MXene-FFPI to different analytes

Analyte	Hg^{2+}	Mg^{2+}	Na^{+}
FSR change (pm)	164	36	45
Sensitivity (pm/μM)	182	40	50

Figure S4 presents the performance of the MXene-FFPI for Hg^{2+} detection in DI water and bottled drinking water. Figures S4a, b show the interference spectra and corresponding FSR responses obtained in DI water, exhibiting a concentration-dependent decrease in FSR from 17.76 to 17.59 nm over the Hg^{2+} concentration range of 0.05–1.0 μM. Figures S4c,d present the interference spectra and corresponding FSR responses obtained in bottled drinking water spiked with different Hg^{2+} concentrations, exhibiting a concentration-dependent decrease in FSR from 17.77 to 17.59 nm over the Hg^{2+} concentration range of 0.05–1.0 μM. As summarized in Table S2, recovery rates ranged from 90% to 109%, with RSD values below 6.95%, confirming the good accuracy and reproducibility of the proposed sensing platform in a representative drinking water matrix.

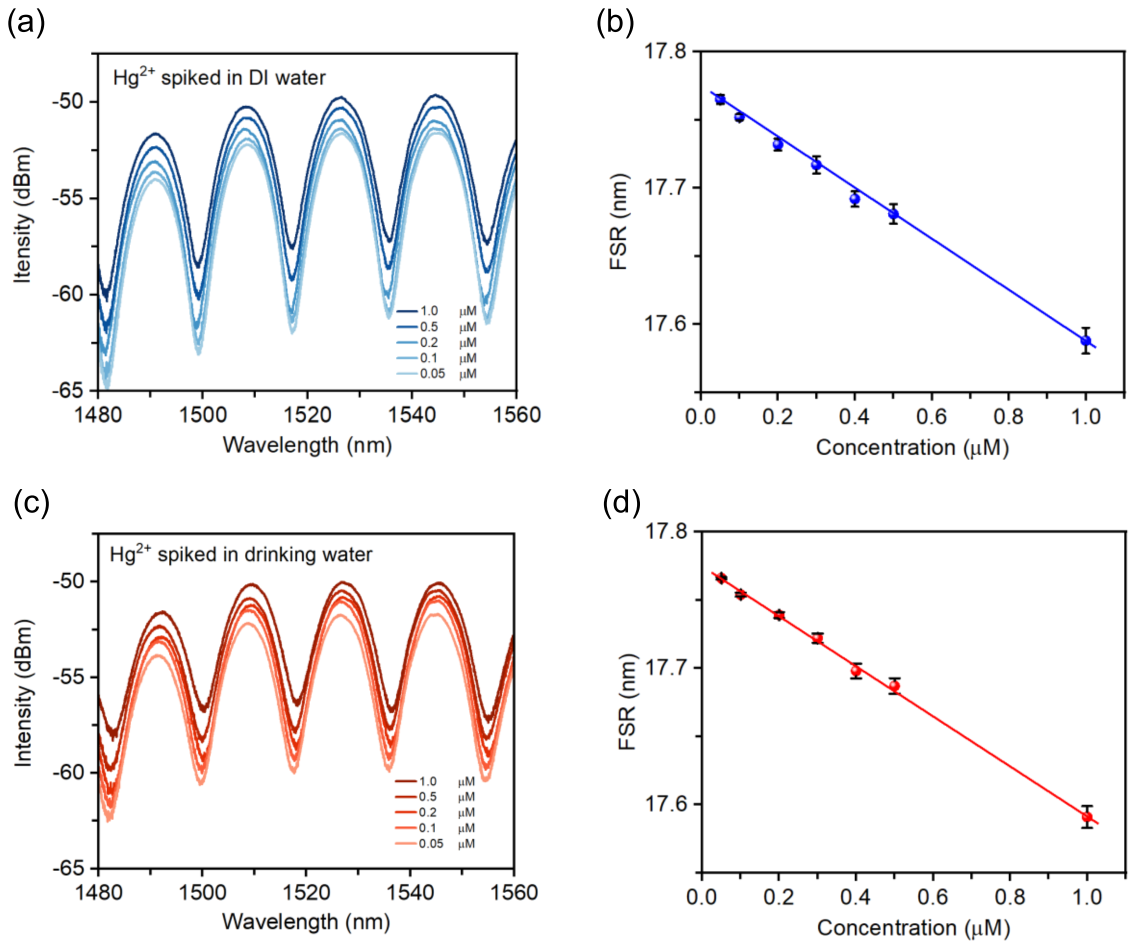

Figure S4. MXene-FFPI for Hg^{2+} detection in DI water and bottled drinking water. (a) Interference spectra of the MXene-FFPI recorded at different Hg^{2+} concentrations in DI water (selected spectra for clarity). (b) FSR as a function of Hg^{2+} concentration in the range of 0.05–1.0 μM for DI water. (c) Interference spectra of the MXene-FFPI recorded at different Hg^{2+} concentrations in drinking water (selected spectra for clarity). (d) FSR as a function of Hg^{2+} concentration in the range of 0.05–1.0 μM for drinking water. Data in (b) and (d) are presented as mean ± s.d.

Table S2 summarizes the recovery experiments conducted to evaluate the practical applicability of the MXene-FFPI in a drinking water matrix. Commercially available bottled natural mineral water was used directly without pretreatment and spiked with Hg^{2+} at concentrations ranging from 0.05 to 1.0 μM. The measured recovery rates ranged from 90% to 109%, with relative standard deviation (RSD) values between 3.87% and 6.95%. These results demonstrate that the proposed sensing platform maintains good accuracy, precision, and analytical reliability for Hg^{2+} quantification in drinking water samples.

Table S2. Recovery experiment data for the detection of Hg^{2+} in drinking water samples

Hg^{2+} added (μM)	Hg^{2+} found (μM)	RSD (%)	Recovery (%)
0.050	0.045±0.003	6.95	90.0
0.100	0.109±0.007	6.56	109.0
0.200	0.189±0.011	5.98	94.5
0.300	0.278±0.015	5.44	92.7
0.400	0.407±0.028	6.88	101.7
0.500	0.468±0.029	6.24	93.6
1.000	0.983±0.038	3.87	98.3

References

[R1] Yu, L.; Bati, A. S. R.; Grace, T. S. L.; Batmunkh, M.; Shapter, J. G. $Ti_3C_2T_x$ (MXene)-Silicon Heterojunction for Efficient Photovoltaic Cells. *Adv. Energy Mater.* **2019**, *9* (31), 1901063.

[R2] Chen, L.; Wakeel, M.; Haq, T. U.; Chen, C.; Ren, X. Insight into UV-induced simultaneous photocatalytic degradation of $Ti_3C_2T_x$ MXene and reduction of U(VI). *J. Hazard. Mater.* **2022**, *430*, 128377.
